# Suprathreshold Approaches to Mapping the Visual Field in Advanced Glaucoma

**DOI:** 10.1167/tvst.12.6.19

**Published:** 2023-06-26

**Authors:** Jonathan Denniss, Allison M. McKendrick, Andrew Turpin

**Affiliations:** 1 School of Optometry and Vision Science, University of Bradford, Bradford, UK; 2 Department of Optometry and Vision Sciences, University of Melbourne, Parkville, Victoria, Australia; 3 Lions Eye Institute, Nedlands, Western Australia, Australia; 4 Optometry, School of Allied Health, University of Western Australia, Perth, Western Australia, Australia; 5 Computing & Information Systems, University of Melbourne, Parkville, Victoria, Australia; 6 Curtin School of Population Health, Curtin University, Perth, Western Australia, Australia

**Keywords:** glaucoma, perimetry, visual field, advanced, suprathreshold

## Abstract

**Purpose:**

Measuring the spatial extent of defects may be advantageous in advanced glaucoma where conventional perimetric sensitivity measurements are unreliable. We test whether suprathreshold tests on a higher density grid can more efficiently map advanced visual field loss.

**Methods:**

Data from 97 patients with mean deviation < –10 dB were used in simulations comparing two suprathreshold procedures (on a high-density 1.5° grid) to interpolated Full Threshold 24-2. Spatial binary search (SpaBS) presented 20-dB stimuli at locations bisecting seen/unseen points until the seen status of all neighbors matched or until tested points were adjacent. The SupraThreshold Adaptive Mapping Procedure (STAMP) presented 20-dB stimuli where entropy was maximal and modified the status of all points after each presentation, stopping after a fixed number of presentations (estimated as 50%–100% of the presentation number of a current procedure).

**Results:**

With typical response errors, SpaBS had worse mean accuracy and repeatability than Full Threshold (both *P* < 0.0001). Compared to Full Threshold, mean accuracy (Full Threshold: median, 91%; interquartile range [IQR], 87%–94%) was slightly better with STAMP for all stopping criteria, although this was not statistically significant until 100% of conventional test presentations were used. Mean repeatability for STAMP was similar for all stopping criteria (*P* ≥ 0.02) compared to Full Threshold (Full Threshold: median, 89%; IQR, 82%–93%).

**Conclusions:**

STAMP accurately and repeatably maps the spatial extent of advanced visual field defects in as few as 50% of conventional perimetric test presentations. Further work is needed to test STAMP in human observers and in progressive loss.

**Translational Relevance:**

New perimetric approaches may improve information available for advanced glaucoma management and may potentially be more acceptable to patients.

## Introduction

Current treatment for glaucoma aims to slow or halt the progression of visual field loss. Determining the efficacy of treatment or the need to modify treatment relies on monitoring the visual field and ocular structures for signs of progressive damage. Current clinical tests used for monitoring the visual field in glaucoma have significant limitations in cases of advanced disease. First, test times increase in advanced visual field loss,^1^^,^^2^ meaning that data quality may be affected by patient comfort or drifts in attention, and tests may be less acceptable to patients unless they provide more useful data.^3^^,^^4^ Second, in areas of damage, the test procedures themselves yield variable results that render the detection of further progression increasingly difficult.^1^^,^^5^^,^^6^

For patients with advanced visual field loss, the preservation of small amounts of remaining visual field may be crucial to their quality of life and daily activities. This means there is a need for improved clinical tests that can be deployed to monitor progression in cases of advanced visual field loss. Such tests should ideally be faster than current tests, thus improving patient comfort and acceptability, and they must yield useful, repeatable information about the visual field.

Most current threshold static automated perimetry (SAP) tests measure sensitivity at a fixed grid of test locations. Given the limited time available to conduct clinical SAP tests, this approach prioritizes estimating sensitivity information at a cost of limited spatial information. Sparse test grids are known to overlook localized defects in early glaucoma,^7^^–^^12^ and it follows that they are likely to also miss changes in the spatial area of defects in progressing glaucoma. Further, in advanced visual field loss, the threshold sensitivity data captured by clinical SAP tests are so imprecise that changes in sensitivity at fixed locations are unlikely to be detectable.^1^^,^^5^^,^^6^ This increase in measurement variability in advanced loss can be attributed primarily to the flattening of psychometric functions, or “frequency of seeing” curves,^13^ which makes precise measurement of thresholds in low sensitivity areas clinically unfeasible.

An alternative approach to testing the visual field in advanced glaucoma may be to obtain measurements at many more spatial locations by spending much less time testing each individual location. Such an approach would sacrifice resolution of sensitivity measurements to gain improved spatial resolution. There are many possible ways this broad approach could be implemented, depending on the desired trade-off between spatial resolution (number of test locations) and sensitivity resolution. For proof of concept, we hypothesized that suprathreshold testing of many locations, chosen adaptively to maximize spatial information within a short test duration may be a useful way to more rapidly test the visual field in advanced glaucoma.

In this study, we use computer simulation to evaluate two new high-spatial-resolution suprathreshold procedures: spatial binary search (SpaBS), in which locations with a different defect status (defect/no defect) are bisected by new test points until a maximum resolution is reached, and SupraThreshold Adaptive Mapping Procedure (STAMP), in which locations are selected for testing in an entropy minimization procedure. These new procedures were compared to an existing procedure with added spatial interpolation to equate resolution across procedures. We hypothesized that the new procedures would enable useful information to be gained about the spatial nature of advanced glaucomatous visual fields when terminated with a significantly reduced number of stimulus presentations.

## Methods

### Data

Empirical data from one eye each of 97 patients with mean deviation (MD) of –10.0 decibels (dB) or worse were used as input to computer simulations. Initial longitudinal visual field data from 139 patients between the ages of 18 and 85 years with primary open-angle glaucoma, normal-tension glaucoma, pigmentary glaucoma, or treated angle-closure glaucoma were downloaded from the Rotterdam Ophthalmic Data Repository.^14^^–^^16^ The dataset exclusion criteria were secondary glaucomas except pigmentary; evidence of standard automated perimetry visual field abnormality consistent with other disease; best-corrected visual acuity worse than 0.3 logMAR; refractive error outside the range of –10.0 to +5.0 diopters (D); cataract surgery in the previous 12 months; previous refractive or vitreoretinal surgery; evidence of diabetic retinopathy, diabetic macular edema, or other vitreoretinal disease; previous keratoplastic surgery; diabetes; leukemia; AIDS; uncontrolled systemic hypertension; or multiple sclerosis or (other) life-threatening disease.^16^ Visual fields were measured using the Full Threshold 24-2 program of the Humphrey Field Analyzer (Carl Zeiss Meditec, Jena, Germany).^16^ Reliability indices were not available for this dataset; therefore, we made the assumption that the included visual fields were considered reliable by the perimetrist and/or reviewing clinician and the collators of the dataset.

**Figure 1.**
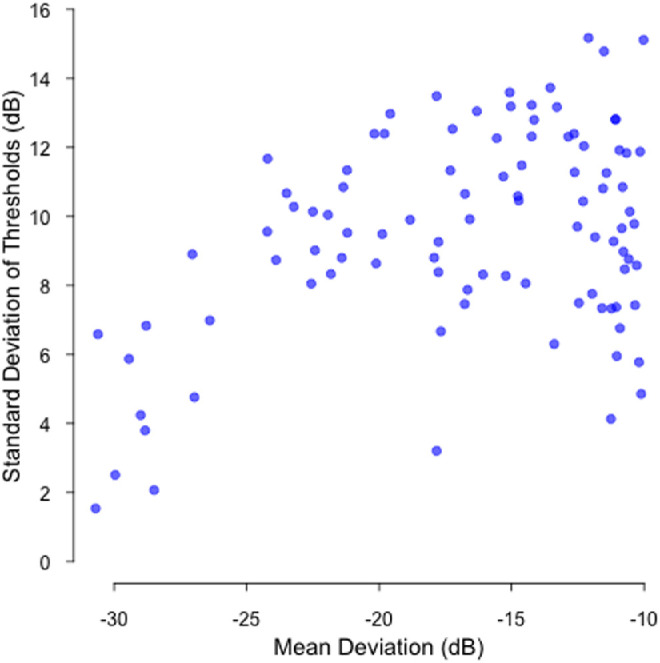
Mean deviation versus standard deviation of thresholds for all 97 included visual fields. The standard deviations of thresholds are provided as a measure of spatial uniformity of defect, as pattern standard deviation was not available. A lower standard deviation indicates a more uniform visual field, such that perimetrically blind visual fields would appear in the *bottom left* of the plot.

The initial dataset was filtered to include only the latest visual field of each patient with a final MD of –10.0 dB or worse. For patients where both eyes reached this criterion, the eye with the worse MD was chosen. One patient with perimetrically blind visual fields was excluded. This resulted in a final dataset of 97 visual fields from 97 patients. Left-eye visual fields were converted to the right-eye format. Figure 1 shows the distribution of MDs and the standard deviations of thresholds (as a measure of spatial uniformity of defect, as pattern standard deviation was not available) for all included visual fields.

As the patient data were on a 24-2 (6° square) grid, spatial interpolation was required to match the higher spatial resolution of the new procedures to be tested. This was achieved using the kriging method previously employed for microperimetry data.^17^^,^^18^ A method similar to that employed previously^17^ was used to determine the parameters for surface fitting by universal kriging. Briefly, surfaces were fit to the data with a variety of range parameters from 0.05° to 3.0° in 0.05° steps. Mean pointwise root mean squared differences between the fitted surface values and measured sensitivities across all patients were measured for each range parameter. Several range parameters achieved equally good performance on this metric, so we chose 1.5°, as it was the smallest range parameter that achieved optimal performance, thus maximally preserving spatially localized information. As per the previous study,^17^ a quadratic trend surface and exponential covariance matrix were used, and the nugget parameter (α) was set to 0. For further details of the fitting method see Reference 17. Patient data were spatially interpolated to a 0.5° square grid for use as input to simulations.

### Baseline Procedure: Full Threshold With Post Hoc Spatial Interpolation

A simple way to obtain higher resolution sensitivity data without the need for new perimetric procedures would be to employ post hoc spatial interpolation to sensitivity data from an existing procedure. Therefore, we simulated Full Threshold with post hoc spatial interpolation and application of a 20-dB cut-off to define locations as seen/unseen as a baseline procedure for comparison. The Swedish Interactive Threshold Algorithm (SITA) Standard procedure commonly employed in clinical practice has test–retest characteristics similar to those of Full Threshold with shorter test times,^19^ but its full details are not available in the public domain. Full Threshold was simulated as previously.^2^^,^^20^^,^^21^ Briefly, Full Threshold is a staircase procedure that presents stimuli in 4-dB steps until the first response reversal, then in 2-dB steps until a second response reversal is obtained. The output threshold is the last seen stimulus at this point. Four primary locations at (±9°, ±9°) are tested first, with the staircase starting from 25 dB. Further locations are then tested through a growth pattern (shown in Turpin et al.^1^) (Fig. 1) in which staircases begin from the end point of their neighbors with a correction for eccentricity. If no response is received to the 0-dB stimulus twice at a given location, that location is assigned 0 dB. In common with the clinical Full Threshold application, any locations whose output threshold is more than 4 dB away from the start point is tested again in the same way, but starting from the first threshold estimate. In these cases, the final output threshold is the last seen stimulus of the second staircase. Additional staircases used by the clinical application of Full Threshold to determine short-term fluctuation are not simulated here. As such, our simulation of Full Threshold uses approximately one more presentation per location than SITA Standard.^1^^,^^22^^,^^23^

The Full Threshold procedure used a 24-2 grid. Upon completion, the same interpolation method described earlier for the empirical data was used to interpolate the output sensitivities to the 1.5° grid. A 20-dB cut off was then applied to the interpolated sensitivities to label locations “defective” or “not defective” for comparison with the new procedures.

### New Procedures

Two new high-resolution (1.5° square grid covering the central 27°) suprathreshold procedures, SpaBS and STAMP, were compared to interpolated Full Threshold 24-2 by computer simulation (see later). Both new procedures presented stimuli only at 20 dB, with the goal of marking each location on the 1.5° grid as either “defective” or “not defective.” We chose 20 dB for simulations to be at the lower end of sensitivity that can be reliably measured by standard automated perimetry in order to target use in advanced glaucoma.^6^ All procedures simulated in this study could alternatively use any other value, depending on the specific goal of investigation, although this would affect results.

**Figure 2.**
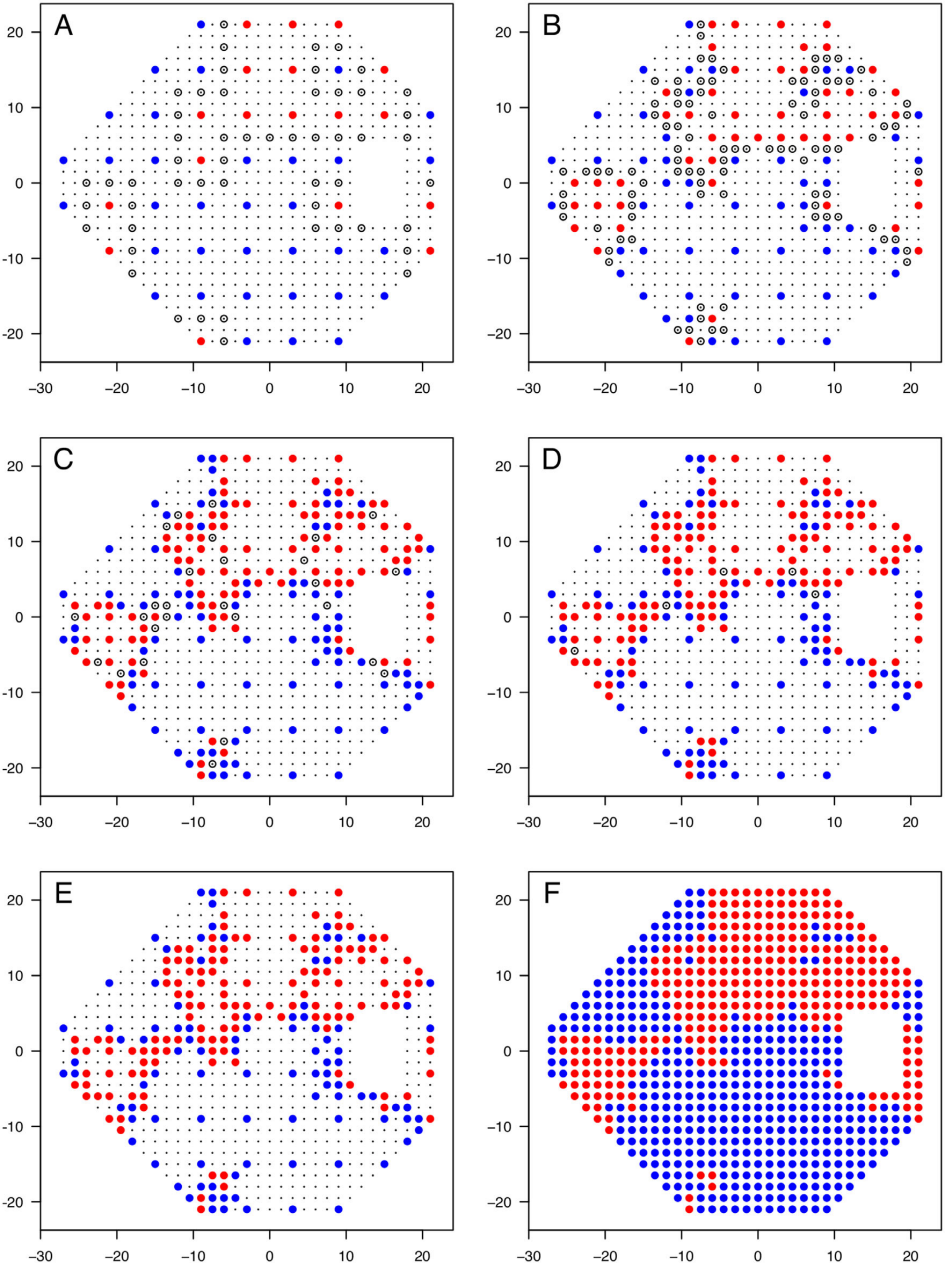
Example of the SpaBS procedure on one visual field. *Red points* indicate defective locations; *blue points*, not defective locations; *smaller black points*, untested permitted locations; and *open circles*, locations chosen for next round of presentations. (A) Seed locations tested first. (B–E) Subsequent rounds of presentations. (F) Final visual field, in which untested locations are interpolated from the tested locations shown in (E).

The SpaBS procedure begins from the 24-2 grid as seed locations. A single stimulus presentation was made at each location, with each location being marked as seen or unseen accordingly. Subsequently, stimuli were presented in the same way at locations bisecting tested locations with opposing seen/unseen status in any cardinal or ordinal direction within √72° (the maximum separation of the seed locations). This process was repeated iteratively until the status of all neighboring tested locations as seen or unseen matched or until tested points were adjacent on the 1.5° grid. This results in an uneven distribution of tested locations across the visual field, with spatially dense testing around the border of scotomas and sparser sampling away from scotoma borders either within large scotomas or in areas of residual visual field. Upon completion of the procedure, locations on the 1.5° grid that were not tested were interpolated from those that were tested to determine their final seen/unseen status. An example of the SpaBS procedure is shown in Figure 2.

The STAMP procedure is an adaption of the SWeLZ (Spatially Weighted Likelihoods in Zest) concept, in which a response at a given location is spread to neighboring locations with a reducing impact by spatial distance.^24^ In the original SWeLZ implementation,^24^ each location had a probability mass function (pmf) over possible thresholds. In the suprathreshold scenario of this study, there are only two possible statuses for a location: defective (D) or not defective (N). When a response (“seen” or “unseen”) is received at a location, the pmf is updated using Bayes rule as

$$\begin{array}{c} P(status|response)=P(status)\times P(response|status)/Z \end{array}$$

where

$$\begin{array}{c} Z=P(D)\times P(response|D)+P(N)\times P(response|N) \end{array}$$

*P*(*status*) is initially set to 0.5 for all locations. If perimetry was a perfect test of vision, then *P*(*unseen*|*D*) = 1 and *P*(*seen*|*D*) = 0 (and vice versa for status *N*). However, perimetry has some response variability, so we allow for this by saying

$$\begin{array}{c} P\left(response|D\right)=\left\{\begin{array}{cc} 1-\propto & \mathrm{if}\;\mathrm{response}\;\mathrm{is}\;\mathrm{unseen}\\ \propto & \mathrm{if}\;\mathrm{response}\;\mathrm{is}\;\mathrm{seen} \end{array}\right. \end{array}$$

and

$$\begin{array}{c} P\left(response|N\right)=\left\{\begin{array}{cc} \propto & \mathrm{if}\;\mathrm{response}\;\mathrm{is}\;\mathrm{unseen}\\ 1-\propto & \mathrm{if}\;\mathrm{response}\;\mathrm{is}\;\mathrm{seen} \end{array}\right. \end{array}$$

At the location of testing, ∝ = 0.01, and then, similarly to SWeLZ,^24^ this ∝ is increased with spatial distance *d* degrees from the test location as

$$\begin{array}{c} \propto \;=0.01+0.6\times \left(1-1/\exp\left(d\times 0.17\right)\right) \end{array}$$

If we define *p_i_* = *P*(*D*) as the probability that location *i* is defective, such that *P*(*N*) = 1 – *p_i_*, then *p_i_* is updated to *u* = *p_i_*(1 − ∝)/(*p_i_* + ∝ − 2*p_i_*∝) for an unseen response, and *s*  = *p_i_*∝/(1 + 2*p_i_*∝ − *p_i_* − ∝) for a seen response.

In order to choose a location in which to present a stimulus, we select randomly from the set of locations on the 1.5° grid that will lead to a decrease in expected entropy over all probabilities of at least 5% of the maximum possible decrease at each iteration. Entropy is a concept from information theory that represents the amount of information inherent to the possible outcomes of a variable. In this case, it relates to how much information would be gained about the visual field by presenting at a particular location. The expected entropy at a particular location is given by

$$\begin{array}{c} H_{i}=-p_{i}\log_{2}u+\left(1-p_{i}\right)\log_{2}s \end{array}$$

and the expected entropy over all locations is calculated as

$$\begin{array}{c} H=\sum H_{all\;locations} \end{array}$$

**Figure 3.**
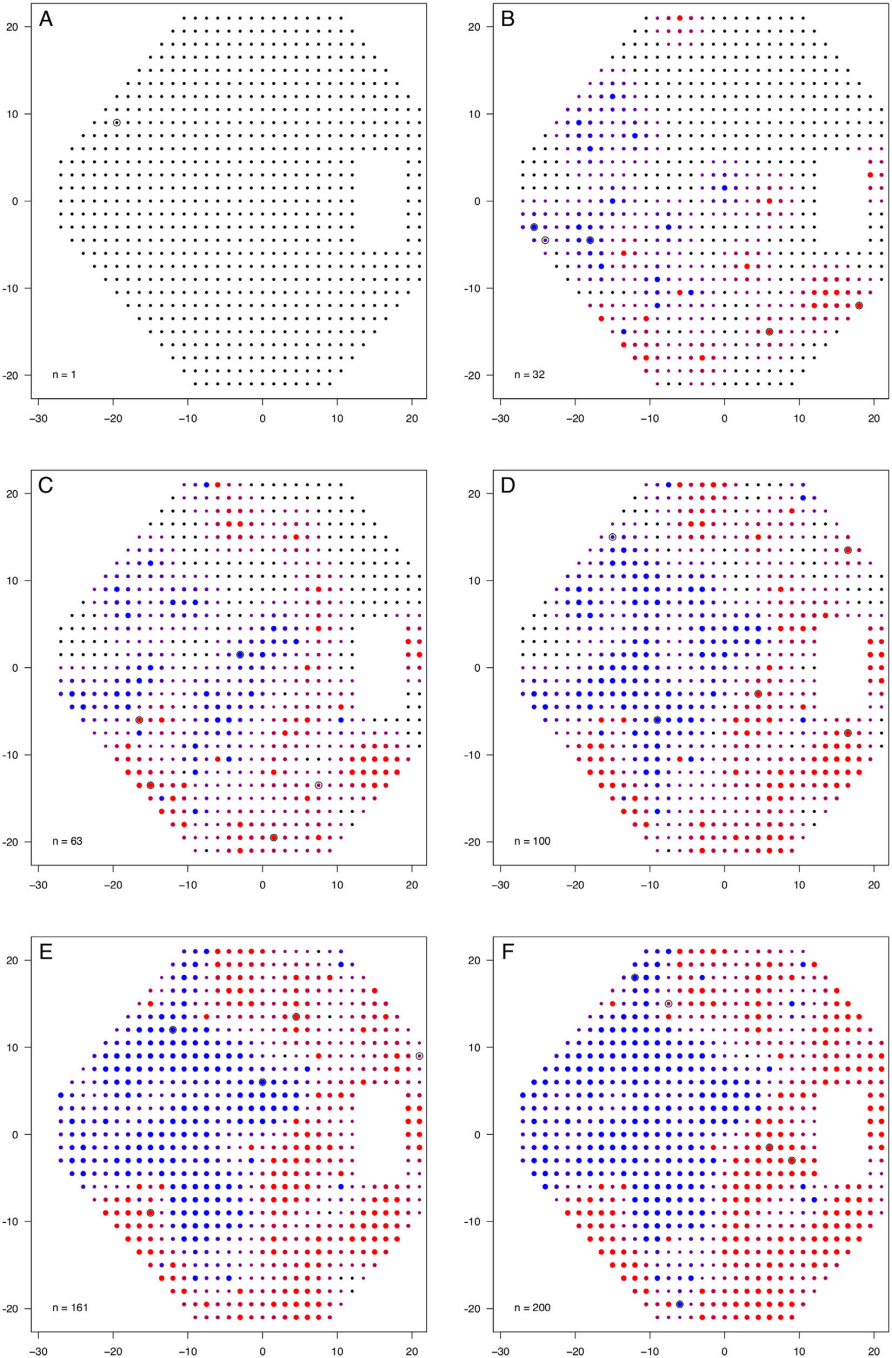
Example of the STAMP procedure on one visual field. The color of points is linearly graded from *red* to *blue* according to the probability that the point is defective (*red* indicates defective; *blue*, not defective). The *smaller black points* indicate untested permitted locations; *open circles*, location of last five presentations. Each “*n* =” gives the number of presentations used to that point. (A) Starting point. (B–E) Subsequent rounds of presentations. (F) Final visual field. In (B) to (F), *point size* is proportional to entropy (arbitrary scale), with *smaller points* having greater entropy (more uncertainty).

The procedure loops, iteratively choosing presentation locations and updating probability values using these rules until a stopping criterion is reached. In this study, the procedure stopped after a fixed number of presentations had been made. At the end of the procedure, locations with *p_i_* > 0.5 were marked as “defective,” and others were marked as “not defective.” An example of the STAMP procedure is shown in Figure 3.

### Computer Simulations

Procedures were simulated using similar techniques to previous studies.^1^^,^^2^^,^^20^^,^^21^^,^^25^^,^^26^ Briefly, the interpolated empirical visual field data acted as input “true” thresholds at every location on the 1.5° grid for simulation. For each visual field location of each simulated patient, the probability of responding to a presented stimulus of intensity *x* was modeled with the function

$$\begin{array}{c} P(response)=1-\mathrm{FN}-(1-\mathrm{FN}-\mathrm{FP})\times G(x,t,s) \end{array}$$

in which *G*(*x*, *t*, *s*) represents the value at *x* of a cumulative Gaussian function with a mean equal to the assumed true threshold *t* and standard deviation *s*. False-negative and false-positive response rates are given by *FN* and *FP*, respectively. The spread of the function (*s*) varied with the input threshold (*t*) according to a function given by Henson et al.,^13^ except that the spread was capped at 6 dB to better match more recent empirical data:^27^^,^^28^

$$\begin{array}{c} s=\min\left(\exp\left(-0.081t+3.27\right),\;6\right) \end{array}$$

Each procedure was simulated 200 times per simulated patient (*n* = 97, therefore 19,400 total simulated visual fields) to generate distributions of output visual fields for comparison.

We compared Full Threshold with SpaBS and STAMP under response error conditions we estimated to be typical for naïve observers, or slightly above average for experienced observers.^29^ The three procedures were simulated with 5% false-positive responses and 3% false-negative responses.

Although SpaBS and Full Threshold have implicit stopping criteria, STAMP does not and would therefore continue indefinitely without a predetermined stopping criterion being imposed. Stopping criteria could be chosen based on entropy (overall or pointwise), but in this study we chose to employ a simple criterion of stopping after a preset fixed total number of presentations across all locations. We simulated STAMP stopping after various numbers of total presentations, chosen to be approximately 50%, 60%, 70%, 80%, and 100% of the median number of presentations we estimated SITA Standard would make on these patients. This was calculated as the median number of presentations made by Full Threshold minus one per location.

In order to evaluate performance under other response error conditions, Full Threshold and the best performing of the new procedures were also simulated with no response errors (false-positive rate and false-negative rate both 0%) and high false-positive errors (false-positive rate 15%, false-negative rate 3%).

### Data Analysis

Conventionally, new visual field test procedures that aim to measure threshold sensitivities at fixed locations are evaluated by the accuracy and precision (repeatability) of output thresholds compared to input “true” thresholds. This form of evaluation is not suitable here, however, because the procedures report only seen or unseen at 20 dB. This results in a binomial distribution over repeated tests that cannot be evaluated by the conventionally used metrics such as standard deviation of repeated thresholds as a measure of precision or repeatability. Accuracy and repeatability of the three procedures were therefore assessed as follows.

As a reference against which to measure the accuracy of the three procedures we applied a 20-dB cut off to the 97 interpolated empirical visual fields, marking every location on the 1.5° grid as “seen” if its interpolated sensitivity was 20 dB or greater or “unseen” if its interpolated sensitivity was below 20 dB. The accuracy of a visual field as measured by one of the three procedures was defined as the percentage of locations in the measured visual field whose seen/unseen status matched that of the reference visual field. The mean accuracy of the 200 repeated visual fields was taken for each patient as the measure of procedure accuracy.

Repeatability of the procedures at a given test location can be assessed by considering the proportion of times that location is designated “seen.” If “seen” designations are denoted by 1 and “unseen” designations are denoted by 0, then the mean response over repeated tests of a perfectly repeatable procedure would be either 0 or 1. Alternatively, a procedure operating at chance level would have a mean response of 0.5. We therefore used the function

$$\begin{array}{c} \begin{array}{c} Repeatability=abs(2\times proportion\;seen\;over\;repeat\;tests-1) \end{array} \end{array}$$

to define pointwise repeatability. This function results in values from 0 to 1, where 0 represents chance repeatability (50% of repeats have the same status, whether seen or unseen) and 1 represents perfect repeatability (100% of repeats have the same status, whether seen or unseen). Any other value for proportion seen results in a value between 0 and 1, being linearly closer to 1 when the proportion seen is closer to either 0 or 1. We then took the mean of these pointwise values across all repeated visual fields to give an overall estimate of repeatability for each simulated patient and procedure.

Finally, we used the number of presentations as a surrogate for test time, as is conventional in computer simulations of visual field test procedures. The SITA Standard procedure widely used in clinical practice uses approximately one fewer presentation per location than our implementation of Full Threshold; therefore, we have taken this into account in interpreting our results. Note that further test time savings are made by SITA Standard over the clinical implementation of Full Threshold through factors that are not relevant here, such as a dynamically adaptive response window and reduction in the number of catch trials.^22^^,^^23^ The mean number of presentations across each of the 200 repeated visual fields was taken for each patient as the measure of procedure speed.

Between-procedure comparisons in number of presentations, accuracy, and repeatability were made using paired Wilcoxon rank-sum tests (paired by patient). Bonferroni corrections were applied as indicated in the Results section to account for familywise error rates when making multiple comparisons. All computer simulations and data analyses were performed in R 3.6.1 (R Foundation for Statistical Computing, Vienna, Austria).^30^

## Results

### Number of Presentations

Full Threshold used a median of 310 presentations per visual field (interquartile range [IQR], 274–335), equating to approximately 258 presentations for SITA Standard. We therefore simulated STAMP stopping after 127, 154, 180, 200, and 255 presentations to simulate approximately 50%, 60%, 70%, 80%, and 100% of the number of presentations made by SITA Standard, erring on the side of slightly fewer. The SpaBS procedure made a median of 284 presentations (IQR, 259–309)—fewer than Full Threshold (*P* = 0.001) but more than the 258 that SITA Standard would be expected to make (*P* < 0.0001). All simulated STAMP stopping criteria except 255 represented statistically significant reductions in presentation numbers over that expected for SITA Standard (all *P* < 0.0001). The number of presentations made by Full Threshold was weakly correlated with the MD of the input visual field (Spearman's rho = 0.33; *P* < 0.001). The positive correlation is driven by lower number of presentations in very advanced loss where most points have sensitivity < 0 dB. The number of presentations made by SpaBS was not correlated with the MD of the input visual field (Spearman's rho = 0.1; *P* = 0.33). Therefore, in our sample, the greatest benefit of STAMP is for visual fields with MDs of approximately −10 to −20 dB.

### Accuracy and Repeatability

**Figure 4.**
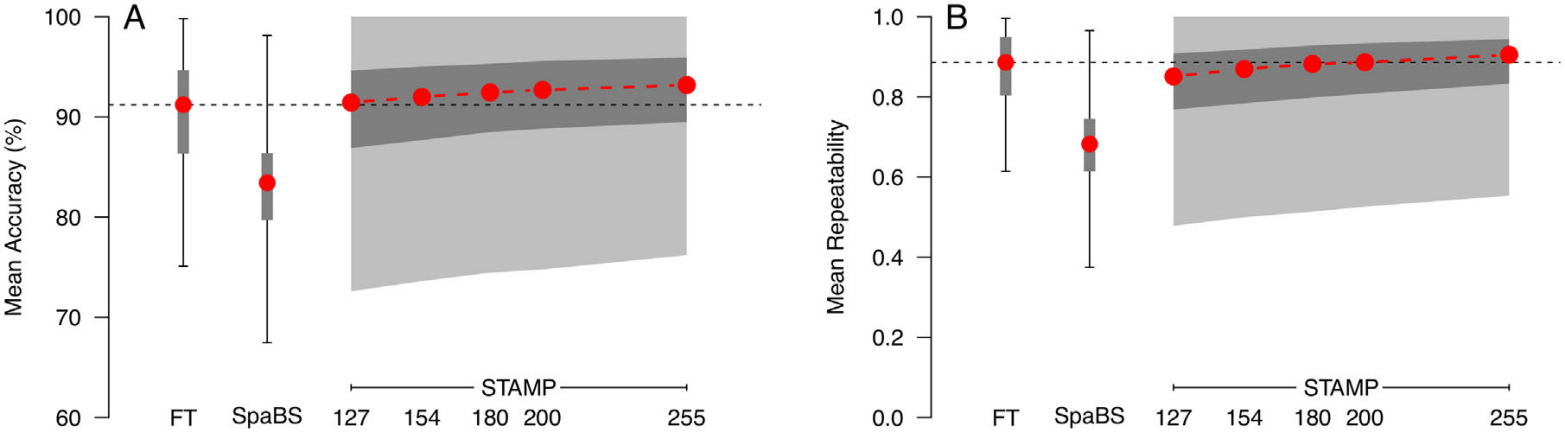
Mean accuracy (A) and repeatability (B) for Full Threshold SpaBS and STAMP with five different stopping criteria. STAMP stopped after a fixed number of presentations as shown on the horizontal axis. *Boxes* and *whiskers* show IQRs and full ranges, respectively, for Full Threshold and SpaBS. For STAMP, *darker* *gray* *shaded areas* show IQRs, and the *lighter* *gray* *shaded* areas show full ranges. *Red points* show medians. *Horizontal dashed lines* show medians for Full Threshold for comparison.

Mean accuracy and repeatability for Full Threshold, SpaBS, and STAMP with all five stopping criteria are shown in Figure 4. As shown in Figure 4, SpaBS was median 7.8 percentage points less accurate (*P* < 0.0001) and median 0.2 less repeatable than Full Threshold (*P* < 0.0001). As expected, both the accuracy and repeatability of STAMP increased as a function of the number of presentations made. The median accuracy of STAMP exceeded that of Full Threshold already when stopping after 127 presentations (∼50% of SITA Standard), although this improvement only reached statistical significance (*P* = 0.008, with statistical significance assumed at *P* < 0.01 accounting for five comparisons) for STAMP stopping after 255 presentations (approximately equal to SITA Standard). The median repeatability for STAMP exceeded that for Full Threshold when stopping after 180 (70% SITA Standard) or more presentations; however, this improvement never reached statistical significance with the stopping criteria tested (*P* = 0.30 for STAMP with 255 presentations). Where Full Threshold repeatability exceeded that of STAMP, this again was not statistically significant with the stopping criteria tested (*P* = 0.02 for STAMP with 127 presentations, *P* = 0.20 for STAMP with 154 presentations).

For these visual fields with advanced loss, the accuracy and repeatability of SpaBS were invariant with the MDs of the input visual field (Spearman's rho for accuracy = −0.10, *P* = 0.32; Spearman's rho for repeatability = −0.11, *P* = 0.27). The accuracy and repeatability of STAMP stopping after 127 presentations were also invariant with the input visual field MDs (Spearman's rho for both accuracy and repeatability = −0.11, *P* = 0.27). For Full Threshold, accuracy and repeatability were both weakly correlated with MDs (accuracy rho = −0.55, *P* < 0.001; repeatability rho = 0.34, *P* < 0.001).

### Alternative Error Conditions

**Figure 5.**
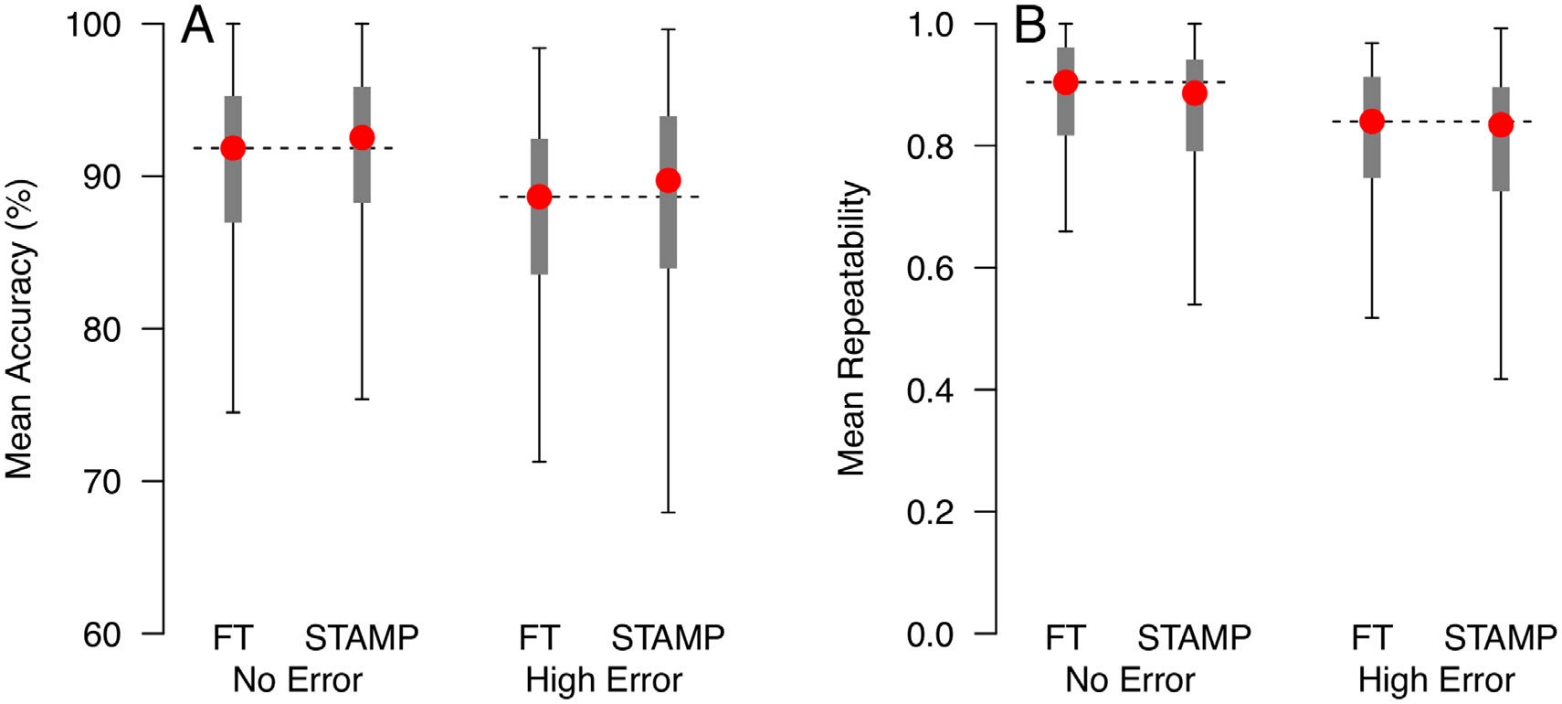
Mean accuracy (A) and mean repeatability (B) for Full Threshold and STAMP stopping after 154 presentations under no-error conditions (0% false-positive and false-negative rates) and high-error conditions (15% false-positive rate, 3% false-negative rate). Plotting symbols are as described for Figure 4. *Horizontal dashed lines* indicate the median performance of Full Threshold under each error condition for comparison.

We chose to simulate STAMP stopping after 154 presentations, approximately 60% of SITA Standard, to compare to Full Threshold under conditions with no response errors (0% false-positive and false-negative rates) and high false-positive errors (15% false-positive rate, 3% false-negative rate). The 154 presentations condition was chosen, as it had statistically similar accuracy and repeatability compared with Full Threshold yet represents a considerable saving over SITA Standard in terms of number of presentations. Results of these simulations are shown in Figure 5.

Under the no-error condition, Full Threshold made a median of 300 presentations per visual field (IQR, 265–330), meaning that the SITA Standard would be expected to make around 248 presentations per visual field. The STAMP procedure simulated therefore represents a median expected saving of 94 presentations (38%, *P* < 0.0001) over the SITA Standard under these conditions. Also under the no-error condition, accuracy and repeatability for STAMP were similar to those for Full Threshold (accuracy median 0.7 percentage points higher, *P* = 0.36; repeatability median 0.018 lower, *P* = 0.14).

Under the high false-positive error condition, Full Threshold made a median of 322 presentations per visual field (IQR, 304–341), meaning that the SITA Standard would be expected to make around 270 presentations per visual field. The STAMP procedure simulated therefore represents a median expected saving of 116 presentations (43%, *P* < 0.0001) over SITA Standard under these conditions. Under the high error condition, accuracy and repeatability for STAMP were again similar to those for Full Threshold (accuracy median 1.1 percentage points higher, *P* = 0.45; repeatability median 0.006 lower, *P* = 0.25).

## Discussion

Visual field test procedures currently used for monitoring glaucoma measure threshold sensitivity at a fixed grid of test locations, thus prioritizing information on the depth of defects over information on the spatial extent of defects. This approach has limitations in advanced loss, where sensitivity information cannot be measured precisely^1^^,^^5^^,^^6^ and no longer contributes to progression detection.^31^^,^^32^ As such, time can be spent obtaining data that are not repeatable and not useful for monitoring disease progression.^31^^,^^32^ Longer tests may also be more tiring and frustrating for patients,^3^ potentially compounding the problem with loss of attention.^33^

In this study, we aimed to assess whether a suprathreshold approach to visual field testing may produce accurate, repeatable measurements of the visual field in advanced cases. Rather than measuring threshold sensitivity, we aimed to measure the area where sensitivity exceeds a predefined cut-off value in higher spatial resolution. In this initial approach, we chose to use 20-dB stimuli as being close to the lower bound of precisely measurable sensitivity in glaucoma^6^ and used a 1.5° grid, four times the spatial resolution of the common 24-2 pattern.

Of our two trial procedures, the conceptually simpler SpaBS procedure made around 10% more presentations than expected for the 24-2 SITA Standard. SpaBS was also less accurate and less repeatable than conventional tests with spatial interpolation applied post-test to equate test location number. There are several methods by which SpaBS could be sped up (such as changing the spacing of the initial seed locations, the resolution of the grid of permitted test locations, or the rules for which neighboring test locations influence each other, or simply terminating after a fixed number of presentations). Such parameterization would speed up the test but would have a detrimental effect on accuracy and repeatability, which already are problematic. We therefore concluded that the SpaBS approach is not worthy of further investigation.

Unlike SpaBS, our second procedure (STAMP) terminates after a fixed number of presentations. Alternatively, STAMP could terminate when entropy reaches a certain level, but this was not tested here. The current termination rule allowed us to titrate the effect of number of presentations on the accuracy and repeatability of STAMP. Median accuracy of STAMP was slightly better than that of Full Threshold/SITA Standard when STAMP made half as many presentations as SITA Standard would be expected to make. The accuracy of STAMP continued to increase with number of presentations permitted, making the improvement over Full Threshold/SITA Standard statistically significant when the number of presentations matched that expected for SITA Standard. Repeatability began to exceed that of Full Threshold/SITA Standard when STAMP made more than 70% of the number of presentations that the SITA Standard would be expected to make. These results suggest that, under typical response error conditions, STAMP can deliver high-resolution spatial measurements of the visual field in 50% to 70% of the test duration of standard tests. We corroborated this finding under alternative (high/zero) response error conditions, with STAMP making approximately 60% of the presentations of SITA, in which cases STAMP had accuracy and repeatability that closely matched those of Full Threshold/SITA Standard.

Here, we have explored suprathreshold methods for altering the spatial sampling of visual fields in advanced glaucoma. There are other published approaches for algorithmically altering visual field spatial sampling, but STAMP may have advantages in truly advanced field loss. One such example is the Gradient-Oriented Automated Natural Neighbor Approach (GOANNA) procedure, a Bayesian procedure that starts with a series of seed locations and then adds additional test locations along the steepest gradients within the visual field.^34^^,^^35^ An alternative approach is the Australian Reduced Range Extended Spatial Test (ARREST), which is not a thresholding algorithm per se but instead a logic for the selection of new test locations when existing locations have been deemed too unreliable to be worth testing.^36^^,^^37^ ARREST commences with the 24-2 test pattern (or other standard pattern). When a location has sensitivity below a critical cut-off value (for example, 17 dB), that location no longer undergoes “thresholding” at the next visit but is only presented a 0-dB stimulus. This allows the presentations saved to be used to test new locations. These new locations are selected based on visual field gradients and undergo thresholding using any standard procedure so existing normative data can be used.^36^ Both GOANNA and ARREST have test times similar to those of existing procedures. GOANNA can be applied at any stage of disease, whereas ARREST is designed to optimize information acquired when there is moderate disease. In truly advanced disease, the suprathrehold nature of STAMP may be preferable for patients, although this requires human testing.

The STAMP procedure shows promise for measuring the visual field in advanced glaucoma; however, the principles of STAMP could be applied with different test parameters for different purposes. For example, STAMP could be applied with a lower intensity stimulus individually determined or appropriate to patient age^38^ as a possible screening test for early glaucoma. Alternatively, STAMP could be applied iteratively with different stimulus intensities, yielding information akin to that of kinetic perimetry. STAMP applied this way has potential advantages over kinetic perimetry: reduced test time, identical psychophysical task to familiar static automated perimetry for the patient, and requiring no further operator training beyond that for conventional static automated perimetry. Further study may also show STAMP to be useful for other diseases affecting the visual field. STAMP is further agnostic to the pattern of test locations used; for example, our simulations already covered the 10-2 area within the 24-2 area.

In this study, we compared STAMP with the SITA Standard, finding that STAMP is capable of mapping the visual field with fewer presentations than the SITA Standard, thereby offering reduced test time. It is pertinent to note that there are already procedures available that reduce test time compared to the SITA Standard. Most notably, SITA Faster reduces test times compared to SITA Standard, but its test–retest variability in areas of low sensitivity is greater than that of SITA Standard (about 1-dB greater test–retest SD, equivalent to about a 4-dB wider test–retest 95% range^39^). This means that it is likely that STAMP would require fewer presentations still to produce results equivalent to interpolated SITA Faster. Further, although SITA Faster does make fewer presentations than SITA Standard, some of the test time gains are made through other measures such as shorter response windows, which could equally be applied to STAMP.

The present study was limited to testing a small number of the possible ways this suprathreshold, high spatial resolution approach may be applied to visual field testing. Further, we tested the procedures on existing perimetric data that were captured with the Full Threshold procedure. This has the potential to influence the results, although this influence is likely to be in favor of Full Threshold and therefore most likely to underestimate the benefits of STAMP. Given that the STAMP procedure differs from the Full Threshold procedure with which the empirical data were collected, such as its higher spatial resolution and fixed stimulus intensity, there may be effects on patient attention, fatigue, and test compliance that are not modeled herein. Such effects may influence the test duration, accuracy, and repeatability of the test and can only be assessed by prospective testing of human observers. We have also tested a limited number of observer variability models; however, the results appear consistent across the three different models tested, so we do not expect significant variation in most human observers. Another limitation is that we do not have access to “ground-truth” high-resolution visual fields. As a surrogate, we have interpolated 24-2 visual fields; however, it is likely that some underlying defects will differ from the interpolation of 24-2 data, and such defects could not be tested herein. A key motivation for the development of STAMP was to enable better spatial description of visual field defects in advanced glaucoma without burdening the patient with lengthy test durations. The additional information that is acquired for increased test locations relative to the 24-2 grid may have clinical utility if implemented in practice.

The finding of good accuracy and repeatability for STAMP with reduced test times compared to current procedures is promising for future clinical use, as these are the underlying factors determining the ability of a procedure to measure progression. Existing progression detection methods would not be directly applicable to STAMP; therefore, further study is needed to develop metrics for progression with STAMP and to evaluate STAMP directly in progressive glaucoma.

In conclusion, we have tested two approaches to high-resolution, suprathreshold visual field testing in advanced glaucoma and compared them to an existing test with added post-test spatial interpolation. The SpaBS procedure did not demonstrate benefits over existing tests and is not worthy of further study. Conversely, STAMP demonstrated significant time savings of 30% to 50% per test while maintaining similar or better accuracy and repeatability compared with current tests. This procedure, therefore, shows promise for use in cases of advanced glaucoma, and further study of its ability to detect progression in advanced glaucoma and other diseases is warranted.
